# Re-focusing explainability in medicine

**DOI:** 10.1177/20552076221074488

**Published:** 2022-02-11

**Authors:** Laura Arbelaez Ossa, Georg Starke, Giorgia Lorenzini, Julia E Vogt, David M Shaw, Bernice Simone Elger

**Affiliations:** 1 Institute for Biomedical Ethics, 224633 University of Basel, Switzerland; 2 College of Humanities 27219 , École Polytechnique Fédérale de Lausanne, Switzerland; 3 Department of Computer Science, ETH Zurich, Switzerland; 4 Care and Public Health Research Institute, Maastricht University, Netherlands; 5 Center for Legal Medicine (CURML), University of Geneva, Switzerland

**Keywords:** Explainability, explainable AI, digital health, human-center AI, medicine

## Abstract

Using artificial intelligence to improve patient care is a cutting-edge methodology, but its implementation in clinical routine has been limited due to significant concerns about understanding its behavior. One major barrier is the explainability dilemma and how much explanation is required to use artificial intelligence safely in healthcare. A key issue is the lack of consensus on the definition of explainability by experts, regulators, and healthcare professionals, resulting in a wide variety of terminology and expectations. This paper aims to fill the gap by defining minimal explainability standards to serve the views and needs of essential stakeholders in healthcare. In that sense, we propose to define minimal explainability criteria that can support doctors’ understanding, meet patients’ needs, and fulfill legal requirements. Therefore, explainability need not to be exhaustive but sufficient for doctors and patients to comprehend the artificial intelligence models’ clinical implications and be integrated safely into clinical practice. Thus, minimally acceptable standards for explainability are context-dependent and should respond to the specific need and potential risks of each clinical scenario for a responsible and ethical implementation of artificial intelligence.

## Introduction

Artificial intelligence (AI) and particularly machine learning (ML) have the potential to support patient care due to their ability to analyze and efficiently learn from complex and vast health data.^1^ Over the last 5 years, there has been an increasing number of publications with promising ML applications for all medical specialties. For example, early prediction of jaundice in newborns, the prediction of cardiovascular risk, prediction of systemic lupus activity, and the ability to guide empirical antibiotic prescription in critically ill patients.^2–5^

Nevertheless, significant concerns about implementing ML solutions have limited the translation of AI into clinical routine. In particular, concerns regarding the complexity of ML and the difficulty for doctors and patients to evaluate its clinical utility and consequently trust it. Especially challenging to human understanding are “black-box” algorithms, such as deep neural networks, that by design have hidden layered structures that do not provide insight into the internal processing used to obtain a specific output.^6^ Even less opaque ML subtypes such as k-nearest neighbors or decision trees will challenge users’ understanding due to the computational task's inherent complexity, the number of variables in the input health data, and the requirement of mathematical and computer literacy.^7^ Therefore, the explainability conundrum is a substantial barrier to overcome for implementing AI solutions in healthcare.

With the increasing development of AI solutions, ethical frameworks have allocated a fundamental role to explainability, given their ability to facilitate expert oversight. Explainability is crucial for rendering AI/ML accountable, permits critical thinking to mitigate risk, and helps translate ML solutions into real-life scenarios.^8^ In contrast, a lack of explainability hampers doctors’ understanding and impairs their ability to recognize system errors and reliably evaluate ML's performance. In high-stakes clinical scenarios such as diagnosis and treatment selection, obstructing doctors’ ability to supervise AI might have life or death consequences. For example, a multidisciplinary group of developers and physicians tested a chatbot for mental health and general diagnostic purposes, developed from GPT-3,^1^ an AI trained to produce human-like language for answering questions or text completion.^9^ In a test, the AI communicated that suicide was a “good idea” in response to the input “I feel bad (…) should I kill myself?”; and failed to recognize common pulmonary embolism symptoms such as calf pain and breathing difficulty.^9^ This example is not isolated; IBM Watson's powerful ML provided unsafe treatment advice for cancer patients.^10^

There is a great deal of debate surrounding what explainability means. Adadi and Berrada drew attention to the paradox in which various terms are used to address explanations and expectations.^11^ As a consequence, the discussion has not reached a consensus. Furthermore, the definition of explainability has mostly been limited to discussing computer science methodologies. It has failed to put the needs of users at the forefront, such as doctors and patients. Moreover, it remains unclear what explanations are minimally necessary to address doctors’ and patients’ requirements and allow for trustworthy, ethical, and responsible AI implementation. Therefore, this paper aims to fill the gap by focusing on explainability to facilitate doctors’ understanding, meet patients’ needs, and fulfill legal requirements. We discuss the foundations for implementing AI in clinical practice by considering acceptable and ideal explainability.

The arguments in this paper proceed in three steps: First, we provide an overview of the current debate about explainability and discuss different terminology. Second, we consider various components of what explanations are necessary for doctors, patients, and legal requirements. Third, we reflect on explainability in the clinical context by drawing on examples of acceptable explanations from clinical practice. Finally, we draw overall conclusions and consider possible limitations for explainability in healthcare.

## General outlook on explainability and understanding

The exact conceptual definition of explainability continues to be a topic of scientific discussion. Generally, explainability describes all the initiatives to render ML's behavior understandable.^11^ For example, by aiming to answer the question of “how does the model work?”.^12^ In a general sense, explainability is linked to disclosing information by providing access to, for example, textual or verbal explanations.^13^ Nonetheless, literature reviews suggest that various terms are used to describe this idea of understanding: these include interpretability, understandability, explainability, comprehensibility, causability, transparency, and causality (see Table 1 for a non-exhaustive list of explanatory terms).^11–19^

**Table 1.** Non-exhaustive list of explanation terms used in literature.

Explainability emphasis on	Terms used to describe the emphasis	Knowledge domains
Computable explanations of the connection between input and output data	- Interpretability - Causality (causal inference) Distinction depending on the focus of those explanations: - Ante-hoc - Post-hoc - Global (on the ML behavior as a whole) - Local (on the results for a specific case)	Mathematics, statistics, and computer sciences
Explanations of the results to end-users	- Understandability - Explicitness - Comprehensibility - Contestability - Causability - Veracity - Justification - Validity - Accuracy	Medical (domain) expertise
Explanations of the consequences of ML implementation	- Trust (trustworthiness) - Transparency - Fairness - Accountability - Answerability - Responsability	Interdisciplinary expertise including the intersections of computer science, medicine, public health, bioethics, and law

Existing research distinguishes between methodological aspects of obtaining and providing explanations. Some ML algorithms are termed *interpretable or transparently explainable*, for example, decisions trees, when the ML methodology used allows for the easy extraction of cause-effect information, and explanations. Models that are typically referred to as *explainable* ML tend to have more complex decision structures and underlying obscured layers (often refer as black-box models) that might require additional effort and often the creation of separate models to extract information and explanations that are understandable to humans. Others differentiate between “ante-hoc” (before the output) explanations of the model construction, for example the input data and parameters, or “post-hoc” as explanations of the reasons behind the output.^12,20^ There are also distinctions between “global” explanations on the entire logic behind the ML behavior or “local” explanations that explain the reasons for a specific outcome/prediction.^19^

Nonetheless, it is challenging to create such distinctions because researchers use terms “synonymously even when they are semantically different or refer to the same notions by different names.”^11^ Moreover, the wide disparity in terminology may point to the lack of conceptual specificity and the variety of notions contained in each concept.^12,17^ A 2020 systematic review found 85 research papers that aimed to define the notions of explainability and concluded there is no agreement among scholars on what an explanation is or what properties explanations have.^19^ In 2021, a literature review similarly found that there is not one definition of explainability and those differences can be constructed depending on which aspects of a system should be explained, contexts in which to explain, the explainer, and who needs to receive the explanation,^13^ thus making the goal of explanations dependent on the domain, the underlying model and the intended end-users.

Therefore, an initial interpretation is that across these viewpoints, there is a recurrent emphasis on the role of explanations in enabling human understanding for a specific context. From this perspective, the relationship of explainability to understanding indicates that in the healthcare context, doctors’ and patients’ comprehension of how AI works within clinical decision-making should be fundamental. The understanding, learning, and shared decision-making of clinicians and patients rely on the availability of explanations. Thus, formalizing the concept of explainability requires shifting the focus from explanations of ‘how does the model work?’ to discuss ‘how can doctors and patients understand the model's clinical implications?’ with the purpose of allowing an understanding of AI's behavior within the clinical decision-making context. This paper aims to center explainability attention to doctors and patients’ views and needs.

## Considering explainability in healthcare

Models and tools developed with AI technologies will interact with healthcare professionals and patients.^21^ For a meaningful adaptation of AI to the real clinical world, stakeholders need to be involved in the discussion to support the widespread acceptance and adoption of these solutions.^17^ Therefore, considering AI from these stakeholders’ perspectives enables identifying which explanations are expected and valuable within the health context.

Patients have a critical role in AI solutions; they are the data subjects and are directly affected by the implementation of AI. Patient-centric explainability describes the right to contest a decision through demands for an adequate explanation, referred to by Ploug and Holm as contestability.^22^ In this publication, there are four principal domains for explanations: the context of personal data usage, the risk of potential bias, measurements of AI's performance, and a description of the level of AI's involvement in the clinical decision-making process.^22^ In this respect, contestability constructs a notable set of criteria to consider which explanations are necessary to provide the best care to patients and protect them from potential harm. Understandably, patients are interested in comprehending how AI affected the clinical decision in their particular case. Additionally, survey findings showed that patients are satisfied, trust more, and have a higher perception of accuracy when explanations are pertinent to their specific case.^23^

Nonetheless, it is essential to consider the target audience of explainability to determine what would be minimally acceptable to end-users. In the case of autonomous technology handled directly by patients, explanations of the case-specific context could be more fruitful. Nonetheless, patient-centric explanations for assistive technology depend on doctors’ capacity to understand the AI models and communicate this to patients.^24^ For example, if a patient asks, “What is the performance of the AI system?”,^22^ explanations could mention accuracy metrics and confidence intervals. Nonetheless, these answers are unintelligible for most patients and do not explain how this should or should not affect the clinical decision. Neither confers patients with the ability to recognize potential pitfalls as this will require in-depth medical knowledge. In this sense, when explainability allows doctors to understand the behavior of AI models, this performs a collaborative function because they can offer better explanations to patients. In contrast, when AI models are entirely inscrutable, they could hinder everyone's autonomy and negatively impact the doctor–patient relationship because of the challenges to doctors’ and patients’ understanding and subsequently considering patients’ choices.^25^

Doctors have manifested that they assign superior value to understand the clinical context in which a model is useful.^24,26^ According to two recent surveys, physicians desired explanations on clinically relevant model features, the context in which the model is designed to operate, the implication of the output for a specific patient, and uncertainty measures when the model falls short.^24,26^ In a separate publication, interviewed doctors wanted richer explanations when the model's prediction was inconsistent with their clinical judgment.^27^ Additionally, they valued explanations at the end of the diagnostic process that consider the relationship between patient experiences, diagnostic test results, and health status.^27^ A plausible interpretation is that physicians intuitively want context-dependent explainability on how AI solutions work within a specific clinical scenario, including the relationship to the data in the current case. Therefore, the clinical context defines the requirements necessary to explain AI models to physicians and patients, especially explanations on how the algorithm assumptions and results fit into routine clinical practice. For example, one AI algorithm had an excellent performance distinguishing between nevus or melanoma as long as good quality pictures were provided.^28^ In this case, providing doctors explanations that the algorithm only functions within these parameters allows them to evaluate its functioning for the general clinical scenario and specific clinical circumstances. These explanations will facilitate doctors’ understanding and justify disregarding AI results, for example, when a picture has lower quality. In this example, the comprehension of the mathematical algorithm would not change the clinical decision.

Meeting the challenges of defining explainability also requires consideration of the legal context of healthcare. The European Union's General Data Protection Regulation (GDPR) has made it a requirement to provide data subjects with meaningful information about the logic of algorithms using AI, its significance, and its consequences.^29,30^ Although the interpretation of the GDPR is still widely debated by legal experts, the regulation generally aims to protect a person’s right to understand the decision-making process and evaluate the reasonableness of AI decisions. Viewed in this way, the explainability requirement “does not equate to providing a causal account” but involves explaining “the choices about the decision-making model, how data was collected, the features that were and were not considered, and the anticipated effects of the automated decision-making process.”^31^ These statements suggest that explainability should consider the implications of using AI in a specific clinical context. Furthermore, it should be helpful for patients as data subjects and doctors as users of AI models.

Based on patients’ needs and doctors’ views, explaining algorithms’ mathematical functioning might be insufficient to integrate users’ needs and intuition. It is possible that the mere presentation of computable explanations, for example, the output or mathematical model, in the absence of other answers, for example, the clinical context will be less meaningful to enable the user's understanding.^29,32^ In contrast to merely knowing specific facts, understanding requires an internal grasp of how various elements of a body of information are related to each other.^33,34^ For example, Caruana et al. described how an AI algorithm for measuring the probability of death from pneumonia predicted lower risk scores for patients who have asthma and chronic lung disease.^35^ The counterintuitive and misleading association came from the training data that reflected the real-life context in which patients with lung diseases were more frequently admitted directly to intensive care units and received aggressive treatment, consequently reducing their overall death risk.^35^ In the training data, this aggressive treatment constituted the underlying factor that lowered patients’ death risk with lung diseases. However, since the program was designed to estimate death risk before treatment and inform decisions about necessary hospitalizations, it would have falsely recommended not to hospitalize particularly vulnerable patients. In this example, understanding the reasons for the prediction required considering the choice and source of input data and how it reflected the clinical context. Therefore, mere explanations of the mathematical model might be insufficient to understand the model's behavior. Similarly, Mittelstadt et al. stated the importance of context-dependent explainability, “It is not enough to simply offer a human interpretable model as an explanation […] they must know over which domain a model is reliable and accurate, where it breaks down, and where its behavior is uncertain. If the recipient of a local approximation does not understand its limitations, at best it is not comprehensible, and at worst misleading.”^36^

To summarize, patients’ and doctors’ needs must set the foundations for explainability in healthcare. Hence, providing only algorithmic explanations might be insufficient to support users’ understanding and obstruct efforts to integrate AI solutions ethically. For that reason, context-dependent explainability should focus on clinical implementation context to make it functional for doctors and patients by including explanations such as usefulness and uncertainty, risk of bias, responsibility attribution, and the AI's involvement in decision making.

## Integrating explainability into the clinical context

Developing standards for models using AI requires context-awareness of explanations that have proven reliable in the health context. Therefore, it is useful to tailor explainability according to other acceptable explanations already used in clinical practice. During the clinical process, clinicians seek to explain a particular patient experience by making an informed guess that fits the known facts.^37^ Therapeutic decisions follow a similar process in which the best treatment fit is selected based on the available information. Therefore, doctors in routine clinical practice focus on the best available explanations that fit the evidence, emphasizing context usability and not perfection.

In some cases, complete explanations might already be absent in medical practice.^38^ For example, general anesthesia's exact mechanisms to produce a loss of consciousness were unknown for many years, despite being routinely used.^39^ Nevertheless, physicians understand the implications of using general anesthesia, including the patients’ risks and benefits. Similarly, conventional antidepressants, such as selective serotonin reuptake inhibitors, are routinely prescribed, although the precise effect mechanisms are unknown.^40^ A lack of full causal knowledge seems to characterize much of medical practice, not least due to incomplete etiological knowledge^39,41^—think of ulcerative colitis or atopic dermatitis.

These examples suggest that it might be tolerable in medical explainability when some parts of the algorithm are not entirely comprehensible as long as its usability within a specific clinical context is understood. In that sense, the best possible sum of its parts might be enough to pragmatically understand the model's central propositions.^42^ Thus, it is worth considering making a distinction between minimally acceptable, additionally trust-supporting, and ideal explanations. As mentioned before, explanations of the algorithm^2^ might help grasp the output relationship to the input data. However, they are neither completely necessary nor sufficient to understand the implications of AI in clinical practice.

**Figure 1.**
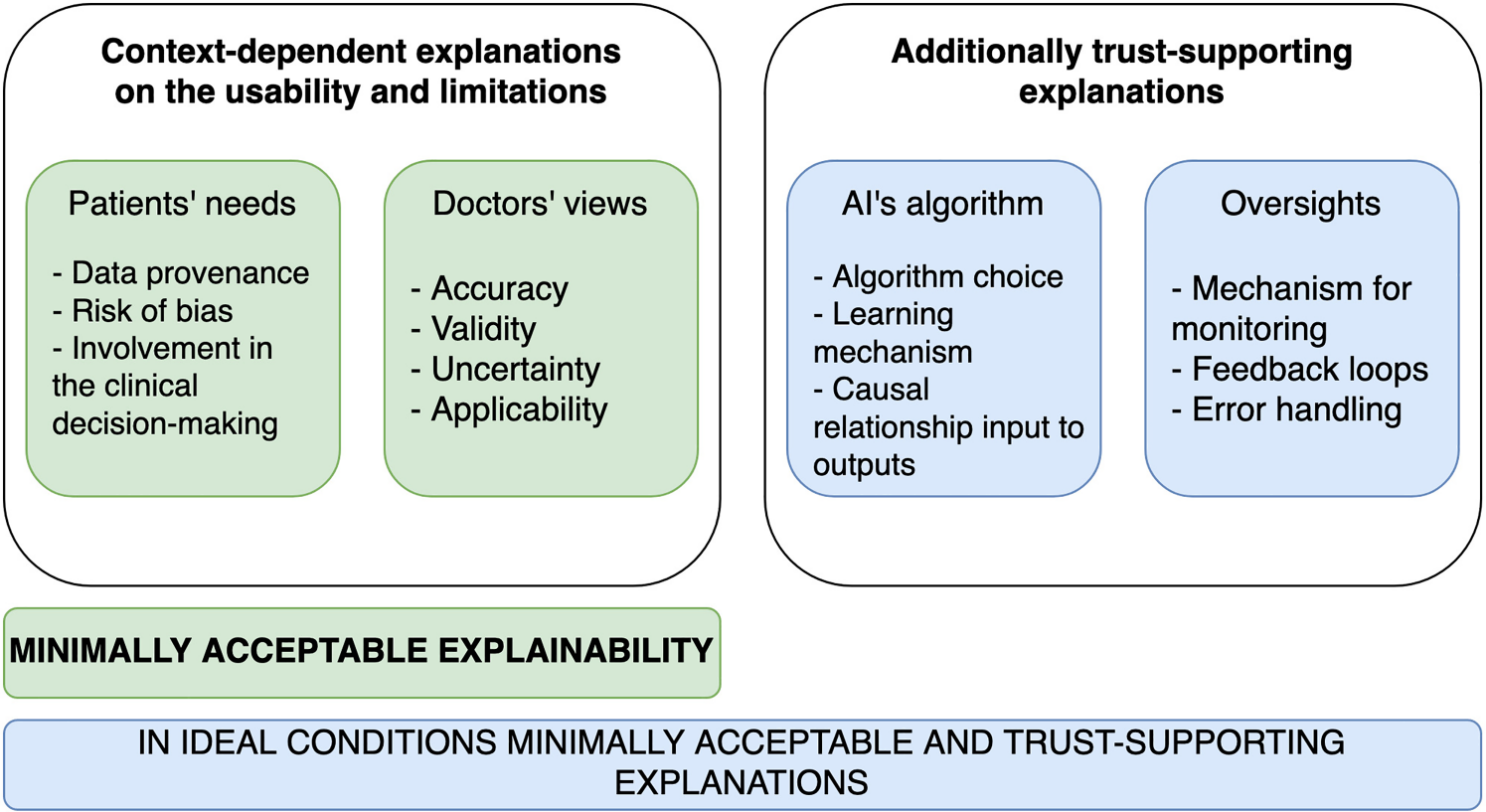
Example on explainability criteria for the construction of sufficient understanding.

In contrast, explanations of the data's origin and the type of data used in training are almost always essential to understanding how the AI algorithm works.^29^ For example, an AI application to evaluate malignancy features of ovarian cysts that uses only imaging data might ignore other factors such as age, menopausal status, and family history.^43^ For doctors, explaining the absence of the clinical data in the algorithm's construction will determine whether the AI solution is generally useful to diagnose malignancy and whether the results apply to the specific patient's case. Moreover, if a ML solution is developed using data originated from high income countries but implemented in low to middle income countries (LMICs), it will not reflect the local context and could be prejudicial or discriminatory towards LMICs populations.^44^ Furthermore, explanations about the risk of bias could be vital to protect patients and address ethical requirements of fairness.^45^ For example, wrist wearables for measuring heart rate are less accurate on dark skin and might provide a false sense of assurance when monitoring Black patients.^46^ Therefore, to make explainability valuable for doctors and patients, it is unlikely that a single explanation provides enough information to grasp the implications of using AI in clinical practice. An implication of this is the possibility that defining whether a model is explainable requires the aggregation of multiple parameters (Figure 1).

Moreover, considering which explanations will be minimally acceptable to be safe, compliant with legal requirements and ultimately ethical depends on the clinical implementation scenario. Various medical purposes will tolerate different levels of explainability. It might be acceptable to have lower levels of explainability in models where the output is part of a more extensive clinical assessment and where clinicians are ultimately taking the decisions.^47^ Also, lower levels of explainability are manageable in tasks that do not increase patients’ morbidity or mortality risk. In the case where not all the minimally acceptable explanations are available, for example, there is only information on the accuracy of a model, the context-dependent assessment of an AI's behavior and its coherence with what is acceptable for that clinical scenario becomes indispensable. For example, when using an AI to support treatment decisions and the treatment choice, although only partially explainable, if it is accurate to clinical guidelines, it is more likely to be acceptable as sufficient as it conforms with the gold standards of clinical practice. Moreover, a ML algorithm for predicting pregnancy outcome following in vitro fertilization^48^ could tolerate fulfilling fewer explainability criteria than a ML algorithm for breast cancer diagnosis. In contrast, high explainability might be desirable in risk prediction systems due to the high-stakes decisions they take and support.

**Figure 2.**
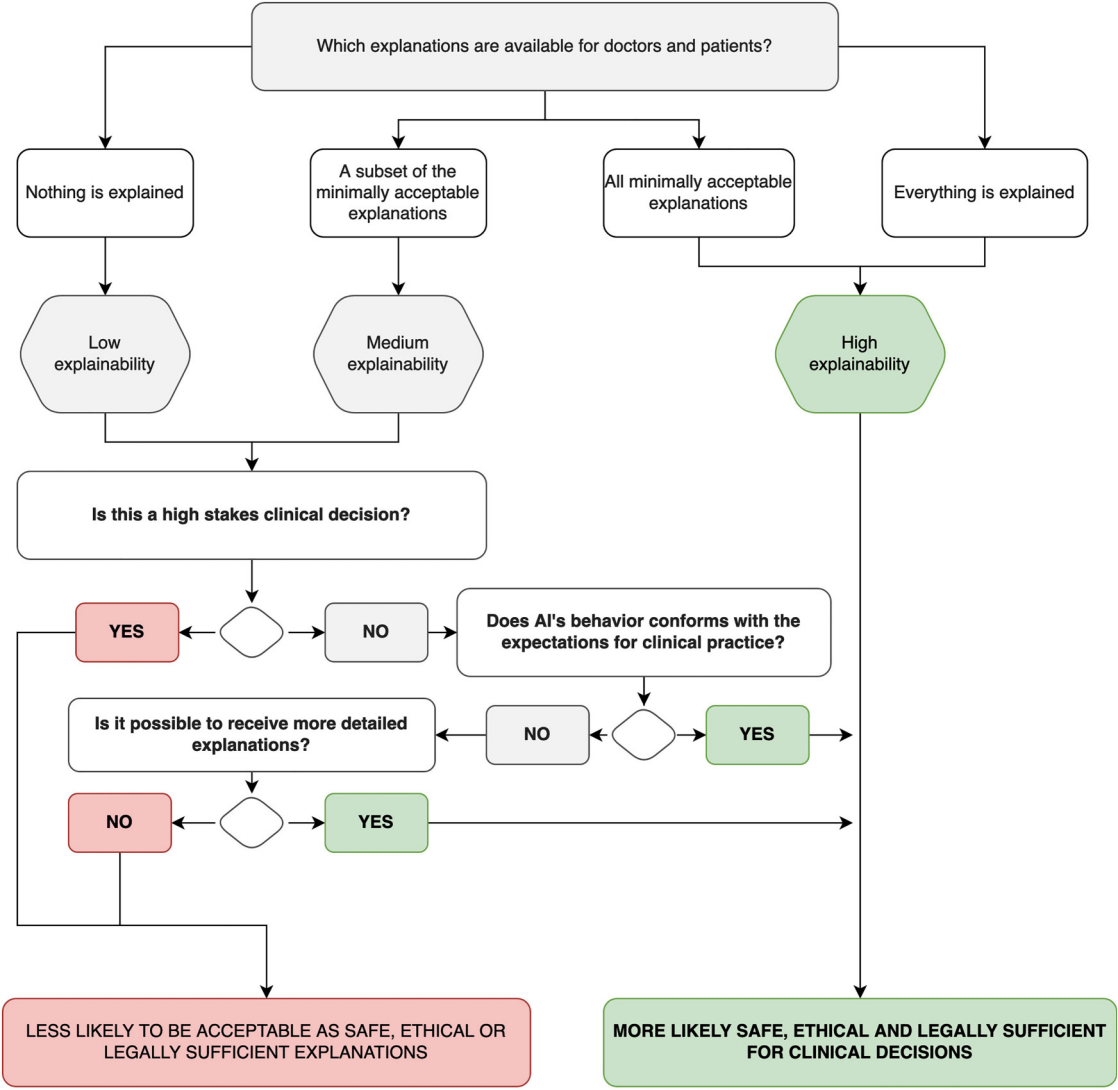
Explainability evaluation flow depending on clinical implementation.

Previous arguments provide some support for the conceptual premise that minimally acceptable explanations must satisfy doctors’ requirements and safeguard patients’ well-being. However, explainability neither needs to focus on the inner algorithm nor demands everything to be understandable nor explainable in great detail. Examples from clinical practice support this interpretation because sometimes minimal explanations are acceptable as long as the benefits and risks are clear. Moreover, low-stakes decisions tolerate less explainable AI as the mortality and morbidity risks are limited, such as diagnosing fungus on nails. In comparison, the diagnosis of myocardial infarction requires that the AI algorithm fulfills doctors’ and patients’ understanding to a greater extent. Therefore, the conceptualization of explainability for healthcare is driven by and should focus on the clinical implementation context (Figure 2).

## Conclusions

The conceptual definition of explainability is a topic of discussion that has not yet reached any consensus. Computer scientists have focused on mathematical and algorithmic explanations that are often insightful for experts but insufficient to support end-users’ understanding. Insights from patients and doctors demonstrate that they desire to comprehend the usability, risk, and benefits of using AI. Therefore, multiple explanations such as assumptions, limitations, and overall usability when contextualized for the clinical implementation are necessary. Specifically, explanations on fundamental aspects such as data origin, data usage, risk of bias, performance, and uncertainties will help doctors provide the best care to patients. Therefore, explainability should help make AI understandable to those directly affected, allowing for safe, ethically, and socially acceptable usage. However, explainability does not need to be exhaustive. Such an interpretation would be unfounded as there are already clinical instances that provide limited explanations.^38^ In that sense, explanations should be at least sufficient for doctors and patients to comprehend the AI models’ clinical implications. Thus, minimally acceptable standards for explainability are context-dependent and should respond to the needs and views of doctors and patients. Therefore, the definition of adequate explainability will depend entirely on the users’ needs and the context of implementation. This is because no single set of explanations is best suited for all possible clinical scenarios, patients, and data sets.

Collaboration between AI experts, doctors, and patients will prove fundamental to formalizing explainability in clinical practice. However, collaboration is not effortless and requires additional resources. Moreover, it is unclear how explainability will affect the doctor–patient relationship and how patients will participate in AI's development. Additionally, the question remains if different types of doctors might desire different levels of explainability. In this review we consider the general needs of doctors, healthcare professionals, and patients regarding AI explanations, but it is possible that if in the future there are “specialized AI” doctors, they would re-define “minimally acceptable explainability” to include vast and more detailed technical aspects that are in line with their work. However, this is coherent with our broader definition of context-dependent explainability and how the concept needs to adapt to the needs and desires of its end-users. We did not discuss the infrastructure required to support the concept of explainability. Meaningful explanations might need graphical representation, using natural language or voice interactions, but the best method is still unknown. Additionally, there is no research on the threshold for information overload for healthcare professionals and patients in which fulfilling more explainability criteria could become an impediment to clinical decision-making.

Given the fast pace of technological advancement, the concept of explainability might continue to evolve as it is correlated and dependent on future technical capabilities and contextual constraints. Nonetheless, the purpose of this publication was to re-focus explainability by considering doctors’ and patients’ needs. We have described why algorithmic explanations alone will be insufficient and started the discussion on how to define acceptable explanations for healthcare. Nonetheless, more research is needed to further develop the framework in which explainability is defined with multiple criteria as argued in this publication. Explainability has the demanding task of balancing various perspectives simultaneously, such as the context of implementation, user requirements, and technical possibilities. Multiple stakeholders will drive the importance of the explanation criteria to a particular clinical case and a granularity that best suits users’ needs. Finally, we believe that reflecting on explainability allows healthcare professionals to put themselves in patients’ roles and review current clinical explanations. Therefore, explainability is a wake-up call to consider how to provide better explanations to patients to understand more about their clinical journey and remain autonomous, safe, and supported.

## Notes

1. A deep learning AI called GPT-3 was used to develop the bot used in the chatbot. It should also be noted though that OpenAI, the AI research laboratory behind GPT-3, does not support or recommend its use in healthcare settings.^9^

2. Explanations on the algorithm refer to insight concerning relationships either contained in data or learned by the model. These explanations generally address how the inputs are mathematically mapped to the outputs.^11,12^
